# Heartbeat Classification by Random Forest With a Novel Context Feature: A Segment Label

**DOI:** 10.1109/JTEHM.2022.3202749

**Published:** 2022-08-29

**Authors:** Congyu Zou, Alexander Müller, Utschick Wolfgang, Daniel Rückert, Phillip Müller, Matthias Becker, Alexander Steger, Eimo Martens

**Affiliations:** Klinikum Rechts der Isar derTechnische Universität München 81675 München Germany; Signal Processing GroupTechnische Universität München 80333 München Germany; Laboratory for AI in MedicineTechnische Universität München 80333 München Germany; Department of ComputingImperial College London 4615 London SW7 2BX U.K.; Fleischhacker GmbH & Company KG 58239 Schwerte Germany

**Keywords:** Convolutional neural network, ECG classification, heartbeat classification, machine learning, mutual information random forest

## Abstract

Objective: Physicians use electrocardiograms (ECG) to diagnose cardiac abnormalities. Sometimes they need to take a deeper look at abnormal heartbeats to diagnose the patients more precisely. The objective of this research is to design a more accurate heartbeat classification algorithm to assist physicians in identifying specific types of the heartbeat. Methods and procedures: In this paper, we propose a novel feature called a segment label, to improve the performance of a heartbeat classifier. This feature, provided by a Convolutional Neural Network, encodes the information surrounding the particular heartbeat. The random forest classifier is trained based on this new feature and other traditional features to classify the heartbeats. Results: We validate our method on the MIT-BIH Arrhythmia dataset following the inter-patient evaluation paradigm. The proposed method is competitive with other similar works. It achieves an accuracy of 0.96, and F1-scores for normal beats, ventricular ectopic beats, and Supra-Ventricular Ectopic Beats (SVEB) of 0.98, 0.93, and 0.74, respectively. The precision and sensitivity for SVEB are 0.76 and 0.78, which outperforms the state-of-the-art methods. Conclusion: This study demonstrates that the segment label can contribute to precisely classifying heartbeats, especially those that require rhythm information as context information (e.g. SVEB). *Clinical impact:* Using a medical devices embedding our algorithm could ease the physicians’ processes of diagnosing cardiovascular diseases, especially for SVEB, in clinical implementation.

## I. Introduction

Cardiovascular disorders cause 30% of the deaths worldwide [1]. The electrocardiogram (ECG) is an important tool for cardiologists to diagnose cardiovascular diseases [2], which can often be regarded as an ECG classification task. Meanwhile, single heartbeat classification based on ECG is also of great importance. Firstly, because ECG consists of heartbeat signals, heartbeat classification can be a foundation of ECG classification. Secondly, a good heartbeat classifier is a better tool to work with than an ECG recording classifier for the following reasons: 1) It can help cardiologists make more sophisticated diagnoses based on certain heartbeats. 2) It is more transparent than the ECG classifier since physicians can verify the results and understand the inference more easily, because it focuses on a smaller segment of ECGs.

In the last decade, many studies utilized machine learning to classify heartbeats. Those machine learning based classifiers are mostly trained with features from the following two categories: 1. medical features [3], [4], such as $pre\_{}rr$, $post\_{}rr$, $local\_{}rr$, $global\_{}rr$, etc. and 2. statistic features in the field of signal processing [3], [4], [5], such as higher-order statistics, wavelet transform coefficients, entropy, and energy density, etc. Besides those two categories, some studies proposed their novel features, such as Sparse Representation [6]. Regarding feature selection, methods like principal component analysis [7], Mutual Information (MI) score [8], and genetic algorithm [9] are employed. As to model selection, mainstream models are: Support Vector Machine (SVM) [3], [10], Linear Discriminants (LDs) [11], XGBoost [12], Neural Network (NN) [13] and Random Forest (RF) [4], [8], which is employed in our work.

In recent years, some researchers also used deep learning to classify heartbeats. Among them, the one-dimensional Convolutional Neural Network (CNN) [14], [15], [16] is more widely used than the Deep Neural Network (DNN) [17]. To take the advantage of the context information around the particular heartbeat, some research use the Recurrent Neural Network (RNN) [18], and some use Echo State Network (ESN), which is less likely to overfit [19].

However, most states of the art methods do not show a very satisfactory performance following the inter-patient evaluation paradigm [20], especially for the heartbeat classes which require context information for that heartbeat, e.g. supraventricular ectopic beat (SVEB). This work focuses on solving this problem.

Cardiological studies suggest that it is impossible to correctly classify some heartbeats only based on the signal of that particular heartbeat itself [2]. Hence some works use RNN to convey temporal context information. However, because the RNN is likely to overfit when data is rare, we choose to use a traditional machine learning model - the random forest, instead of a deep learning model like the RNN. To convey context information without applying RNN, we proposed a novel context feature, called a segment label, for each heartbeat.

A segment label is provided by a pre-trained ECG recording classifier. The classifier predicts the ECG label based on the ECG segment including that particular heartbeat and its surrounding heartbeats. The label summarizes a longer period of the ECG characteristics and implies a cross-heartbeat feature. Therefore, it could provide context information around this particular heartbeat, and serve as a feature for further heartbeat classification. To our knowledge, this is the first work using a deep learning model to generate context features for the training of a heartbeat classifier.

The objective of this work is to design a better heartbeat classification algorithm, especially better on SVEB, with a novel feature: a segment label.

## II. Material

We used three datasets to build and validate our methods. Among them, dataset A is used to train the heartbeat classifier, and hence to validate the heartbeat classification performance. Dataset B/C is used to train two ECG recording classifiers separately. The ECG recording classifier will be employed as one important part of the whole algorithm. We will describe its integration to the method in Section III.

### A. Dataset A

We use a widely-used heartbeats classification dataset - MIT-BIH Arrhythmia [21] to validate our methods. This dataset contains 48 half-hour excerpts of two-channel ambulatory ECG recordings. In total, there are 109,449 heartbeats in 15 sub-classes. And according to ANSI/AAMI EC57:1993/(R)2008 recommendation [22], 15 sub-classes are grouped into 5 classes: normal sinus node (N), ventricular ectopic beat (VEB), supra-ventricular ectopic beat (SVEB), fusion heartbeats (F), unknown beat type (Q). As recommend in [23], four recordings with pacemaker are dropped, and the rest are separated into 22 train recordings (DS1) and 22 test recordings (DS2) in advance, as shown in Table 1.

**TABLE 1:** The Train and Test Recording ids in MITBIH Arrhythmia Dataset, This Division is Following the Recommendation From [23]

	Recording ids
DS1	101, 106, 108, 109, 112, 114, 115, 116, 118, 119, 122, 124, 201, 203, 205, 207, 208, 209, 215, 220, 223, 230
DS2	100, 103, 105, 111, 113, 117, 121, 123, 200, 202, 210, 212, 213, 214, 219, 221, 222, 228, 231, 232, 233, 234

### B. Dataset B

This will be further employed as a pre-training dataset. It is based on the PhysioNet/CinC Challenge 2017 training dataset [24] containing 8,528 ECG recordings. They are processed by the zero-mean unit-variance filter, Then the dataset is augmented by 2,000 additional 10 seconds ECG segments, as well as 2,000 noisy ECG segments. Those noisy ECGs are made by time-reversing the existing 284 noisy ones in the dataset. See more details about the generation of those data in [25]. Their ECG labels are shown in Table 2.

**TABLE 2:** Description of Abbreviation of ECG Labels, Which are Also Used as Segment Labels

Abbreviation of ECG recording labels	Description
N	normal sinus rhythm
A	atrial fibrillation
O	other cardiac rhythms
~	noise segment
AF	atrial fibrillation
IAVB	1st degree AV block
LBBB	left bundle brunch block
NSR	sinus rhythm
RBBB	right bundle brunch block
VE	ventricular ectopic beats
STD	sinus tachycardia depression
STE	sinus tachycardia elevation

### C. Dataset C

This will also be further employed as a pre-training dataset. The dataset consists of 6,877 ECGs from PhysioNet/CinC Challenge 2020 training dataset released at a competition event [26]. Their ECG labels are shown in Table 2.

## III. Methodology

**FIGURE 1.**
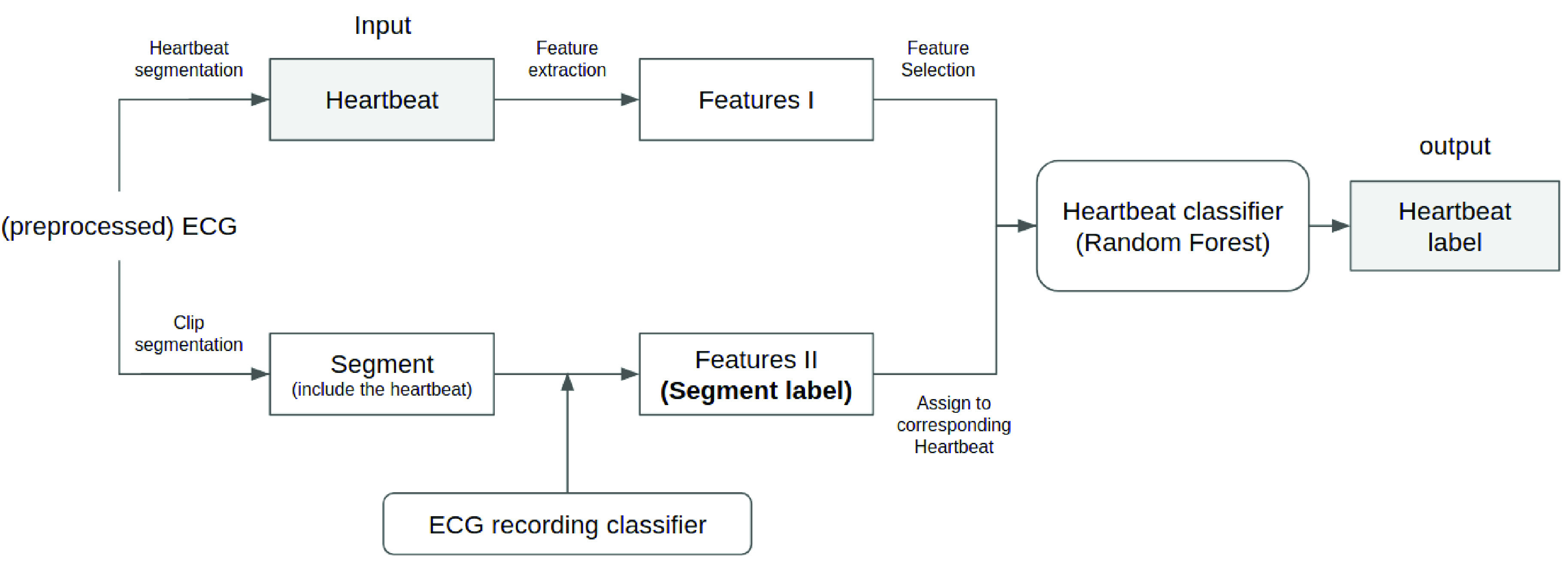
Illustration of the overall methodology.

The overall process of the algorithm is shown in Fig. 1. First of all, preprocessed ECG are cut into continuous heartbeats and ECG segments in parallel. Then, the heartbeats are used to extract features (Features I), while the ECG segments are used to infer the segment labels (Features II) by an ECG recording classifier, and the label is assigned to each heartbeat within the exact segment as a new feature of those heartbeats. Finally, Features II, combining with via Mutual Information score selected Features I, are employed to train a heartbeat classifier.

**FIGURE 2.**
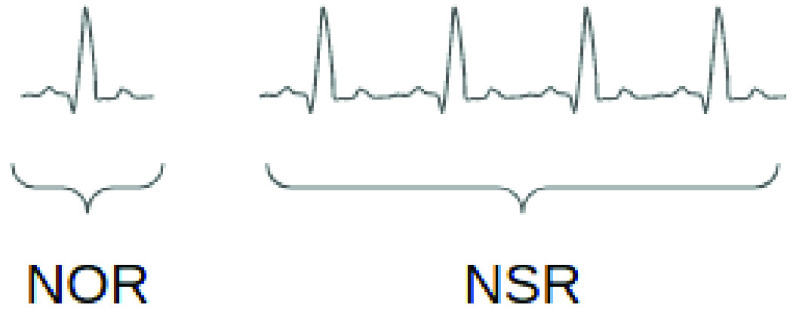
Difference between heartbeat classification and ECG classification. Left side is a single heartbeat with heartbeat label NOR(normal hearbeat). Right side is a period of ECG with ECG label NSR(normal sinus rhythm).

Therefore, two kinds of classifiers evolved within the process. One is a heartbeat classifier of our target. Another one is an ECG recording classifier, as shown at the bottom of Fig. 1. ECG recording classifier is a long-term signal classifier, which takes a period/clip of ECG as input rather than a single heartbeat. Thus, training an ECG recording classifier requires a different type of annotated dataset, compared to training a heartbeat classifier. Specifically, the objects annotated by the labels are not at the same level between heartbeat classification and ECG recording classification, as shown in Fig. 2. In our case, dataset A is employed as a heartbeat-level dataset to train the heartbeat classifier, and dataset B/C is employed as a recording-level dataset to train the ECG recording classifier.

A detailed introduction of the method is in the following:

### A. Data Preprocessing

The signal is pre-processed by resampling the signal to 150 Hz. We also need some fiducial points of ECG for the calculation of some features. R peaks are directly provided from the dataset. Other fiducial points, such as P peak, the start of QRS complex, end of QRS complex, R peak, and S peak are calculated by a heuristic mathematical method same as [8]. Due to space limitations, no detailed introduction is provided here.

### B. Feature Extraction

The features consist of two parts. One is the conventional feature, which is some human-designed feature of that heartbeat, similar to [8]. Another is the segment label.

#### 1) (Part I) Conventional Features

**FIGURE 3.**
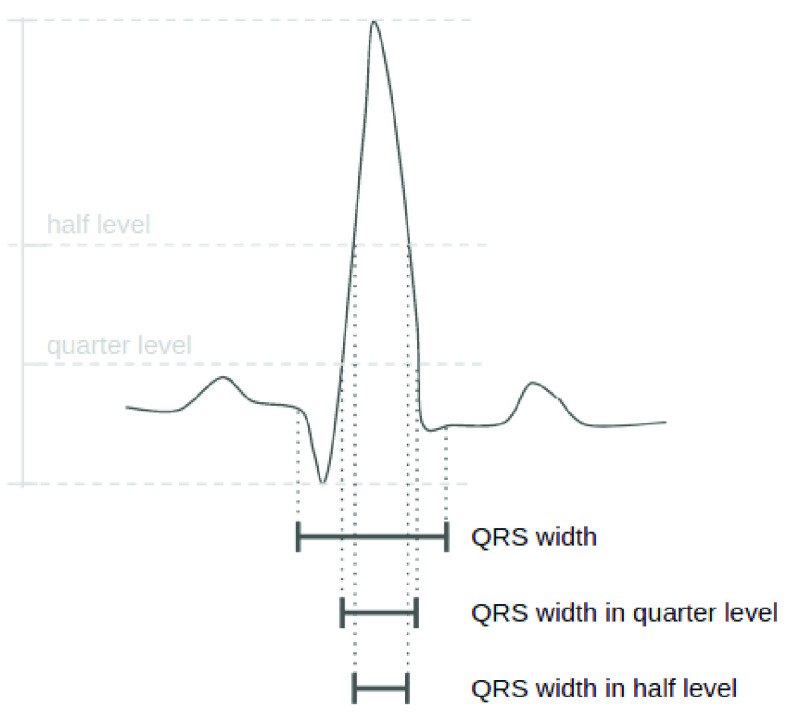
Illustration of QRS width, QRS width in half level, and QRS width in quarter level of one heartbeat. The definition is originally from [8], here half level and quarter level is calculated between the highest signal strength and lowest signal strength of QRS complex.

In the following, the signal of each heartbeat is defined as the ECG signal on the first lead between −250ms and 250ms centered on its R peak:

• **RR interval features (6 features)** $rr$: The current RR interval, which means the number of samples between the R peak of this heartbeat and the previous heartbeat. $pre\_{}rr$: previous RR interval. $post\_{}rr$: post RR interval. Those three features are widely used for machine learning based heartbeat classification; the ratio between $pre\_{}rr$ and $rr$, as well as $post\_{}rr$ and $rr$; $t_{rr}$: t-statistic of $rr$, with the standard error calculated from the last 32 $rr\text{s}$, same as [8].
• **Medical morphology features (12 features)** The amplitude of P wave, Q wave, R wave, S wave, the difference between the amplitude of P and Q wave, Q and R wave, R and S wave, and the distance between P peak and the start of QRS complex. Those 8 features are from [27]; The distance between Q and S peak; Width of QRS, and in half ( $QRSw2$) and quarter level ( $QRSw4$), as defined in [8] and shown in Fig. 3. Additionally, local normalized [8] versions of those 12 medical morphology features are also added to the feature set. They are named as “ $\ldots \_{}norm$”.
• **Mathematical signal morphology features (48 features)** Kurtosis and skewness of five equally length parts of the heartbeat signal as [3]; The same Discrete Wavelet Transform (DWT) coefficients of the heartbeat signal as [3]; and the same Hermite Polynomials Transform (HPT) coefficients of the signal as described in [28] (named as $hbf\_{}i$ for i-th coefficient).

Following previous work [8], we use Mutual Information (MI) score as the feature selection method in our research. Mutual information is a score that measures the information dependence between the features and labels, which indicate the importance of each feature. We rank the features based on their MI scores from high to low, then only select the top-n features. The same proposed number of features $n_{f}$ as in [8], which is 6, is used. The selected features are $QRSw2\_{}norm$, $QRSw4\_{}norm$, $rr\_{}norm$, $post\_{}rr/rr$, $hbf\_{}6$, and $QRSw2$.

#### 2) (Part II) Segment Label

A segment label is a novel feature, a discrete multi-class label. It is a word as a categorical variable. It is acquired from a pre-trained ECG classifier, and used as an additional context feature of the heartbeat. More specifically, the direct output of the pre-trained ECG classifier, which we call it ECG recording classifier, is a probability distribution over several possible ECG classes. We then take the most likely ECG class, namely the ECG class with the highest probability, as the segment label.

**FIGURE 4.**
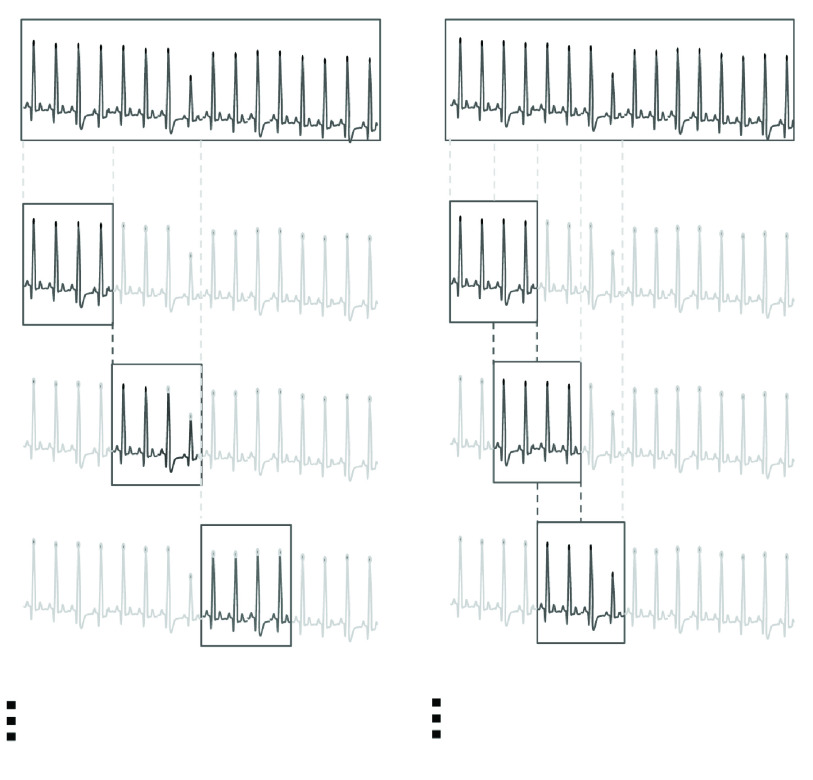
Illustration of ECG segmentation without overlap (left) and with half overlap (right). The upper one are the original ECG signals, and the rests are ECG segments got from the records.

To get the segment label, first of all, the ECG of each patient will be divided into several continuous segments, as shown on the left side of Fig. 4. The concrete length of each ECG segment is determined by the pre-trained ECG classifier. As a comparison, we also tried a different division strategy which is overlapping a half, shown at the right side of Fig. 4. The comparison results is announced in Section IV-C.

Then this pre-trained ECG classification model will predict the label for these ECG segments after they have been preprocessed in the same way as the ECG classifier requires. The label will be assigned to each heartbeat in this segment as the segment label.

To further use segment label as a feature of heartbeats for heartbeat classification, we need to encode it first. We use two ways to encode segment labels. If there is no overlap, we use the label ID as the new feature. If there is overlap, the segment labels will be transformed into a binary string with the length of the count of different possible predictions of the ECG classification model. Each bit of this binary string means whether this heartbeat has that label as the segment label or not.

**FIGURE 5.**
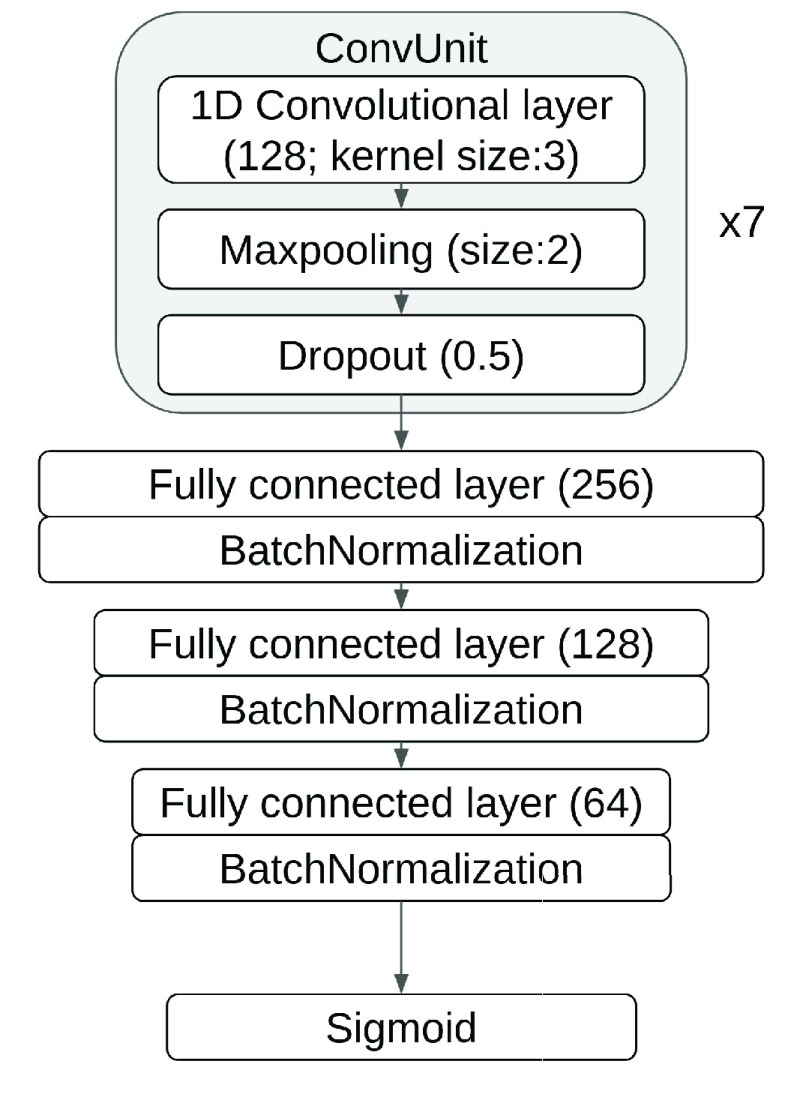
Model architecture of ecgclf_c, which is a ECG recording classifier in Fig. 1 serving as a segment label extractor.

As Convolutional Neural Network (CNN) is widely used as the ECG classifier [14], [15], [16], we tested two kinds of CNN to assign the segment label to each heartbeat in our research. One is a novel CNN framework designed and trained by us, named as *ecgclf_c*. Another one is a ResNet framework from [25], named as *ecgclf_b*. This one serves as a comparison with our novel framework. The following introduces the details of those two segment label extraction models:

2a)
$ecgclf\_{}b$ [25]: It is a ResNet model consisting of 16 1-dimensional ResNet blocks and a softmax last layer. The loss function is categorical cross-entropy. It is trained on dataset B with Adam optimizer. We refer to [25] for additional details.
2b)
$ecgclf\_{}c$: It is a CNN framework designed by us. It has 7 ConvUnits and 3 fully connected layers as shown in Fig. 5. The ConvUnit has a convolutional layer with 128 filters and filter size as 3, a maxpooling layer with pooling size as 2, and a dropout layer with dropout rate as 0.5. Each fully connected layer is followed by a batch normalization mechanism [29]. Finally, the model is covered with a sigmoid layer. Here we did not choose the softmax as the last layer because the ECG label from the dataset could be multiple for some ECGs. The loss function is binary cross-entropy, and the model is optimized by adam optimizer with a learning rate of 0.001.

This segment label extraction model is trained on dataset C. We applied two steps of data preprocessing to the ECG signals from dataset C. One is using two consecutive median filters of 200ms and 600ms to remove the baseline of the ECG signal, similar to [3]. Another is normalizing the signal strength within each record. Besides that, we also cut the signal into 60 seconds if it is longer than 60 seconds, or padded it into 60 seconds with zeros if it is less than 60 seconds. We only took lead-II of the ECG and the signal is resampled to 360 Hz, hence the input has 21600 samples and only one channel ( $x_{seg} \in \mathbf {R}^{21600 \times 1}$). In our case, we used 80% of the data to train the model, 10% to validate it, and 10% to measure the performance of this ECG classification model. We train the model for 35 epochs. The learning rate and the number of epochs are tuned on the validation dataset. We choose the label with the highest prediction score as the model predicted label.

### C. Heartbeat Classification

Random forest is used to classify the heartbeats with $m$ features including features after feature selection and the segment label.

Random Forest is a bagging method of the decision trees. $n_{d}$ decision trees are trained on the same size bootstrap training set. In each split-step of training the decision tree, a subset of $m'$ features out of $m$ features is used to choose the best split variable. The prediction of the random forest model is an aggregation of predictions of every decision tree based on votes counting. Namely, the label with the highest votes is the prediction of the random forest.

In our case, we set $n_{d}=200$, so the random forest has 200 decision trees. We set $m'=\lfloor \sqrt {m}\rfloor $. And the often-used gini function is applied as the function to measure the quality of a split when training each decision tree. Gini-function is defined as follows, where $p_{i}$ stands for the sample probability of class $i$ in each group after the split:

\begin{equation*} Gini=1-\sum ^{c}_{i=1}\left ({p_{i}}\right) ^{2}.\tag{1}\end{equation*}

The random forest is trained with balanced sample weights. The balanced sample weights are inversely proportional to the class frequencies of the input data.

### D. Performance Metrics

To get a more balanced measurement of each targeted heartbeat class, we use the macro-averaged F1-score as the key performance metric of heartbeat classifiers.

Macro-averaged F1-score is defined in the following formula, with $f_{1}^{i}$ means the F1-score of class $i$, and $N$ means the number of classes:

\begin{align*} marco-f_{1}=&\dfrac {1}{N}\sum ^{N}_{i=0}f_{1}^{i} \tag{2}\\ f_{1}^{i}=&2 \cdot \dfrac {p_{i} \cdot r_{i}}{p_{i} + r_{i}}.\tag{3}\end{align*}

In (3), $p_{i}$ (means precision) and $r_{i}$ (means recall) are calculated as true positive(TP)/positive(P) and true positive(TP)/(true positive(TP) + false negative(FN)) for class $i$, respectively.

## IV. Results

### A. Segment Label Quality

The quality of the segment label affects the performance of the heartbeat classifier. As the performance of the ECG recording classifier determines the quality of the segment label, We regard macro F1-score of the ECG classifier as the segment label quality. Namely, the segment label quality from *ecgclf_b* and *ecgclf_c* are 0.72 and 0.70 respectively. The detailed performance of *ecgclf_b* are shown in [25] and detailed performance of *ecgclf_c* are shown in Appendix Table 7.

**TABLE 3:** Confusion Matrix of $RFS_{b}$

		Predicted				
		F	N	Q	SVEB	VEB
Actual	F	5	376	0	0	7
	N	11	43717	0	443	88
	Q	0	7	0	0	0
	SVEB	0	314	0	1441	82
	VEB	0	355	0	16	2842

**TABLE 4:** Confusion Matrix of $RFS_{c}$

		Predicted				
		F	N	Q	SVEB	VEB
Actual	F	68	294	0	0	26
	N	28	43474	0	605	152
	Q	0	6	0	0	1
	SVEB	0	229	0	1452	82
	VEB	31	138	0	14	3038

**TABLE 5:** Performance of Proposed Methods With Different Combinations of of ECG Classifier and ECG Segmentation Method. The Numbers are F1-Scores

Model name	N	VEB	SVEB	macro-avg F1-score
$RFS_{b}$	0.98	0.91	0.77	0.53
$RFS_{b}\_{}overlap$	0.98	0.89	0.72	0.52
$RFS_{c}$	0.98	0.93	0.74	0.58
$RFS_{c}\_{}overlap$	0.98	0.91	0.61	0.56

**TABLE 6:** Performance Comparison of the Proposed Methods and Other Similar Works. (For the Computational Cost, N Means the Number of Samples)

Work	Num. features	Computational cost	ACC	N	VEB	SVEB	SVEB	
				F1-score	F1-score	F1-score	Ppr	Sen
Chen et al. [32]	33	$O(n)$	0.93	0.97	0.77	0.33	0.38	0.30
Mondejar-Guerra et al. [3]	104	$O(n)$	0.94	0.97	0.94	0.60	0.50	0.95
Saenz-Cogollo et al. [8]	6	$O(nlogn)$	0.96	0.98	0.91	0.73	0.68	0.79
Dias et al. [10]	10	$O(n)$	-	0.97	0.92	0.56	0.40	0.93
The proposed method ( $RFS_{b}$)	7	$O(nlogn)$	0.96	0.98	0.91	0.77	0.76	0.78
The proposed method ( $RFS_{c}$)	7	$O(nlogn)$	0.96	0.98	0.93	0.74	0.70	0.79

**TABLE 7:** Detailed Performance of ecgclf_c on Every Classes. The Meanings of Those Abbreviations are Explained in Table 2

Label	Precision	Recall	F1-score
AF	0.89	0.95	0.92
IAVB	0.87	0.79	0.83
LBBB	0.76	0.72	0.74
NSR	0.68	0.72	0.70
PAC	0.61	0.48	0.54
RBBB	0.70	0.67	0.68
VE	0.74	0.85	0.79
STD	0.77	0.67	0.71
STE	0.29	0.40	0.33

### B. Hyperparameter Tuning

**FIGURE 6.**
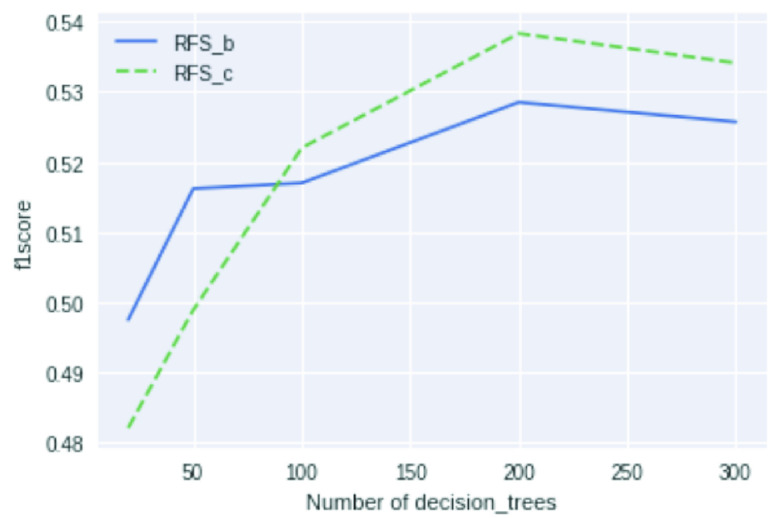
Hyperparameter tuning for number of decision trees based on leave-one-patient-out cross-validation on macro-averaged F1-scores of two proposed models, $RFS_{b}$ and $RFS_{c}$, which are introduced in Section IV-C.

We tune the hyperparameters by “leave-one-patient-out” cross-validation like in [30], in which training heartbeats from a specific recording will be excluded as the validation dataset in each round. Fig. 6 shows the cross-validation hyperparameter-tuning for the number of decision trees $n_{d}$ based on macro-averaged F1-score.

### C. The Proposed Method Performance

We call the model trained with the segment label given by *ecgclf_b* as $RFS_{b}$, means **R**andom **F**orest with **S**egment label extracted from $ecgclf\_{}{\textbf {b}}$, and the model trained with the segment label provided by *ecgclf_c* as $RFS_{c}$.

The models are implemented with scikit-learn framework [31]. The training time for those two proposed models $RFS_{b}$ and $RFS_{c}$, on a machine with Intel(R) Core(TM) i7-9700K CPU @ 3.60GHz, 32GB RAM and a GeForce RTX 2080Ti as the single GPU, are 56.1 seconds and 55.8 seconds, respectively. Those training time are not including the time of training the ECG classification model which extract segment labels.

Table 3 and Table 4 show the confusion matrix of proposed models $RFS_{b}$ and $RFS_{c}$, respectively. It can be observed that the performances on F and Q classes are still not optimal, because of the serious data imbalance problem.

To compare the effect of different segment label extraction methods, different combinations of ECG classifiers and ECG segmentation methods are tested. As shown in Table 5, the overlap mechanism does not contribute to the performance.

It can be observed in Table 5 that $RFS_{b}$’s performance on SVEB is better than others. Meanwhile, $RFS_{c}$’s performance on VEB is better than others. $RFS_{c}$ has the best overall performance by achieving the highest macro-F1-score. This is probably because the performance of VEB label, which has more samples, contributes more to the overall performance.

So we propose $RFS_{b}$ and $RFS_{c}$ as our two new heartbeat classification methods.

## V. Discussion

Table 6 shows the performance comparison between our models and models from other works following the same inter-patient paradigm and the AAMI guidelines. Our models perform the best for SVEB with little cost to the performance of VEB, and our models have the same overall accuracy and F1-score on N as the best existing models. Comparing within our models, $RFS_{b}$ is better on SVEB while $RFS_{c}$ is better on VEB. To the best of our knowledge, there is no work that achieves a higher F1-score on SVEB than the proposed $RFS_{b}$ model. Compared to the work from Saenz-Cogollo [8], which could be considered as the basis of our work, our models achieve better SVEB performance without a drop in performance for other labels. This is proof that the segment label information could help the machine learning model to make better decisions for the heartbeat labels which requires the context information of the ECG.

Unlike other end-to-end heartbeat classifiers, our work not only uses the heartbeat-annotated dataset, but also tries to transfer some knowledge from another dataset. Unlike regular transfer learning, we train an ECG classifier from ECG annotated dataset and then transfer this knowledge from the ECG classifier to a heartbeat classifier via segment label. That is also the main novelty of this paper.

The proposed method could also be applied to recorded ECGs in real-time. The ECG recording classifier and heartbeat classifier will work simultaneously to label the heartbeats every 60 seconds automatically. The advantage of our method is that it has better performance in detecting SVEB, which is also a challenging task for physicians, while not scarifies overall performance.

The success of this approach opens a gate for heartbeat classification to use the knowledge of ECG recording classification. There is much more annotated data for ECG recording classification available to the public. Those data are also a treasure for learning about heartbeat classification. So the question of “how to use this information” deserves more research. Furthermore, The result of this work might not only be bound to the field of ECG analysis. ECG is similar to periodic signal data, and each heartbeat is a rough replicated period of this signal. This work indicates that the class of the whole signal can be a good hint for identifying the correct class of the periods which compose it.

Obviously, the correctness of the ECG classifier is important for this proposed method. In this work, we only tested one novel but simple CNN framework, and a published ResNet model as a comparison. This is just a starter for applying segment labels. Building a better single-lead ECG classifier could be the future work. More advanced architecture such as EfficientNet [33], SE-Net [34], bi-directional RNN [35], or Transformer [36], could be used. Besides that, we simply assign the same segment label to each heartbeat within the ECG segment in this work, whereas in theory, each heartbeat contributes differently to the ECG class. If we could use some kind of attention mechanism to assign this context feature differently, the benefits of segment labels might be greater. Furthermore, current SVEB detection in this work is based on a single lead, meanwhile, algorithms based on 12-leads might show larger improvement.

## VI. Conclusion

In this research, we propose a random forest method to classify heartbeats. This random forest uses features concatenated by two parts. One part is some proposed features from other works as introduced in Section III-B, and we use mutual information scores as the basis for the feature selection. The second part, called the segment label, which is new in this field, is provided by a pre-trained CNN ECG classifier. The proposed methods use this segment label to transfer the context information, that the heartbeat classifier should know about, to the heartbeat. It worked as intended and achieves good results.

With this method, we obtained two heartbeat classification tools that are better at Supra-ventricular ectopic beats(SVEB) detection and also perform well in other classes. We are in the early stages of using this segmented label feature, but we have still made significant improvements. This is proof that it will be very promising to continue research on how to utilize ECG classification to improve heartbeats classification.
